# Quantum free-electron laser oscillator

**DOI:** 10.1038/s41598-026-45068-1

**Published:** 2026-03-30

**Authors:** Peter Kling, Enno Giese

**Affiliations:** 1 German Aerospace Center (DLR), Institute of Quantum Technologies, 89081 Ulm, Germany; 2 Technische Universität Darmstadt, Fachbereich Physik, Institut für Angewandte Physik, 64289 Darmstadt, Germany

**Keywords:** X-rays, Quantum optics, Free-electron lasers, Theoretical physics

## Abstract

If the quantum mechanical recoil of the electron due to its scattering from the undulator and laser fields dominates the dynamics, a regime of the free-electron laser emerges where quantum effects lead to a drastic change in the radiation properties. However, the large interaction length required for a single-pass quantum free-electron laser impedes the experimental realization. The quantum free-electron laser oscillator, proposed in the present article, is a possible scheme to resolve this issue. Here we show that this device features a photon statistics that is closer to a coherent state in comparison to existing classical free-electron lasers. The device can be even operated in such a way that a sub-Poissonian statistics is obtained. Beside the benefit of demonstrating this pure quantum effect, the narrowing of the photon distribution implies reduced intensity fluctuations of the emitted radiation, which in turn lead to decreased noise in imaging experiments or to an enhanced sensitivity in interferometric applications.

## Introduction

The quantum free-electron laser^1–15^(Quantum FEL) is a proposed regime of FEL operation where improved features of the emitted radiation are expected. So far, the research focused on single-pass Quantum FELs with the drawback that the required undulator length is rather large and ultimately reaches a fundamental limit that is determined by the counteracting effects of multiphoton transitions, spontaneous decay, and space charge^12^.

We therefore propose a *Quantum FEL oscillator*, where the emitted radiation of many consecutive electron bunches is stored in a resonator and that can be operated in the low-gain regime with a drastically reduced undulator length compared to high-gain, single-pass FELs. Indeed, the quantum regime requires a high quantum mechanical recoil that translates to an FEL wavelength in the x-ray regime, where up-to-date no suitable high-quality resonators exist. However, in recent years a lot of research was devoted to (classical) x-ray FEL oscillators^16^ (XFELO) based on Bragg diffraction from crystals^17^ and substantial progress was made in this and closely related fields^18–24^.

**Fig. 1:**
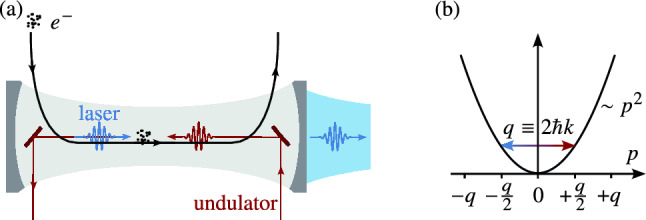
Basic scheme of a Quantum FEL oscillator: In panel (a), the basic elements of the setup are sketched. Bunches of relativistic electrons subsequently interact with the counterpropagating undulator and the copropagating laser field. In the quantum regime of the FEL, we require a high quantum mechanical recoil $q\equiv 2\hbar k$, generated by an optical undulator^4,6,12^ with decreased wavelength. In an oscillator, the laser field is stored and amplified during many round trips in an optical cavity with only a fraction of the light being coupled out. For x rays, the cavity mirrors rely on Bragg scattering from crystals^16^. In panel (b), we illustrate the microscopic mechanisms of a Quantum FEL in the co-moving Bambini-Renieri frame^25,26^. In contrast to the continuous electron trajectories in classical FELs, the longitudinal momentum $p$ of an electron changes by discrete steps^11^ separated by the recoil momentum $q$. Energy-momentum conservation enforces discrete resonances at multiples of $q/2$. For a high recoil and an initial momentum distribution sharply centered at $p=q/2$, we obtain Rabi oscillations between $q/2$ and $-q/2$ with one photon being emitted into the laser mode or re-absorbed by the electron.

In the classical limit^27–32^ many photons are emitted into or absorbed from the laser field^33^ and the discrete nature^34–37^ of the electron dynamics is washed out^38^. However, for a large quantum mechanical recoil $q\equiv 2 \hbar k$ single-photon processes dominate^11^ and the electron occupies only *two* resonant momentum levels, namely $p\cong q/2$ and $p-q \cong -q/2$. We consider the Bambini–Renieri frame^25,26^, where the wavenumbers of the wiggler and laser mode are equal, that is $k_\text {W}=k_\text {L}\equiv k$, and the motion of the electrons is non-relativistic, that is, the kinetic energy follows the non-relativistic dispersion relation. Scattering between the two resonant momenta occurs in agreement with energy-momentum conservation, see Figure 1 (b). This definition of the Quantum FEL^11^ can be quantified by the condition $\alpha _n \equiv g\sqrt{n}/\omega _\text {r}\ll 1$ for the quantum parameter $\alpha _n$, where *g* denotes the coupling strength of an electron of mass *m* to the fields, *n* is the number of laser photons, and $\omega _\text {r}\equiv q^2/(2m\hbar )$ defines the recoil frequency. Moreover, we require that the initial momentum distribution of the electrons, $\rho =\rho (p)$, has to be centered around $p=q/2$ and that its width $\Delta p$ has to be small^3,11^, that is $\Delta p< q$.

In an oscillator configuration sketched in Figure 1 (a), many electron bunches, each consisting of *N* electrons, are injected with a rate $1/\tau _\text {inj}$ and interact subsequently with the fields inside a cavity which simultaneously stores and damps the generated light field^39^. Thus, we identify two contributions to the laser field dynamics, that is (i) Rabi oscillations during the flight time *T* of electrons through the undulator with the momentum-dependent Rabi frequency^11,40^

1 $$\begin{aligned} \Omega _n(p) \equiv \sqrt{g^2(n+1)+\omega _\text {r}^2\left( \frac{p}{q}-\frac{1}{2}\right) ^2}\ \ , \end{aligned}$$

and (ii) cavity damping which is characterized by the quality *Q* of the cavity. With the help of standard methods from laser and micromaser theory^39,41–45^, summarized in the Supplementary Information, we derive the formal expression

2 $$\begin{aligned} P_n=\prod \limits _{n'=1}^n\left[ \theta ^2 \int \!\!\text {d}p \,\rho (p)\, \text {sinc}^2\left( \Omega _{n'-1}(p)T \right) \right] \end{aligned}$$

for the photon statistics $P_n$ at steady state, up to a normalization constant. Here we have introduced the pump parameter $\theta \equiv gT\sqrt{N_a}$ as well as the inverse loss parameter $N_a\equiv NQ/(\omega _\text {L}\tau _\text {inj})$ with the laser frequency $\omega _\text {L}=ck_\text {L}$ in the laboratory frame. Compared to its classical counterpart we obtain a narrowed photon distribution in the quantum regime ranging from super- to sub-Poissonian behavior. This narrowing implies reduced intensity fluctuations of the emitted radiation and by that an increase of the signal-to-noise ratio in imaging schemes or to an enhancement in sensitivity in interferometric applications.

A shorter undulator length for an oscillator facilitates possible Quantum FEL experiments, for example with respect to the implementation of the necessary optical undulator^4,6,12^, since the focal area and pulse duration of the required high-power laser are limited. However, similar to a single-pass Quantum FEL there are tight bounds on the electron beam quality, especially for the energy spread and the beam emittance. Indeed, the emittance of electrons from (photo-)cathodes cannot fall below an intrinsic thermal limit^46^. However, advanced schemes like for example ultracold electron sources^47,48^, that use ionization from ultracold atoms have the potential to provide the required beam quality in the future. Electron bunches from plasma wakefield acceleration^49^ may also meet these requirements, but injecting them at sufficiently high repetition rates needed for the operation of an oscillator remains a serious challenge^50^. Similarly, providing high-power laser pulses for the optical undulator with a rate that matches the injection of electrons is a major obstacle for an experimental implementation.

## Results

In the following we briefly compare the properties of the Quantum and the classical FEL^51–55^. For the time being, we restrict ourselves to the small-signal limit, that is $gT\sqrt{n}\ll 1$ in the quantum regime, leading to a change of the photon number which scales linearly with the initial photon number *n*. A Gaussian approximation^42^ of the photon statistics (see Supplementary Information) yields the expression^56^

3 $$\begin{aligned} \sigma ^2\cong \frac{1}{\delta }\, \end{aligned}$$

for the Fano factor^45^ $\sigma ^2\equiv \text {Var}(\hat{n})/\langle {\hat{n}}\rangle$, where $\delta$ denotes the relative deviation $\delta \equiv (\mathcal {G}-\omega _\text {L}\tau _\text {inj}/Q)/\mathcal {G}$ of the losses $\omega _\text {L}\tau _\text {inj}/Q$ from linear gain $\mathcal {G}\equiv (gT)^2N$ at $p=q/2$. The FEL is operated above threshold and in the small-signal regime if $0<\delta \ll 1$, resulting in a value for $\sigma ^2$ that is larger than unity. Hence, the Quantum FEL shows a super-Poissonian behavior in the small-signal limit.

**Fig. 2:**
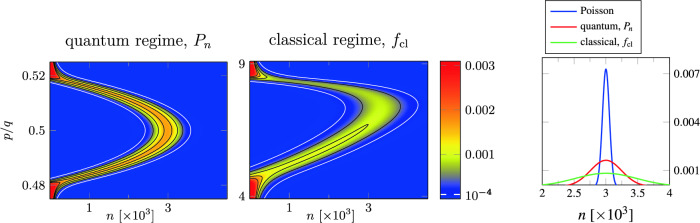
Steady-state photon statistics $P_n$ of an FEL in the quantum regime (left) with $\omega _\text {r}T=20$ and the corresponding distribution function $f_\text {cl}$ in the classical regime (middle) with $\omega _\text {r}T=0.2$, both depending on the photon number $n$ and the momentum $p$ of a peaked initial momentum distribution $\rho (p')=\updelta (p'-p)$. In order to compare quantum with classical regime we have chosen in both cases the same values for the relative deviation $\delta =\delta ^{\text {(cl)}}=0.05$ of losses from gain and the same mean photon number $\langle {\hat{n}}\rangle {=} 3\cdot 10^3$ (for resonance). This choice of parameters brings us to $N_a=2\cdot 10^4$ in the quantum and $N_a=1.5\cdot 10^3$ in the classical case, respectively, for the inverse loss parameter $N_a\equiv NQ/(\omega _\text {L}\tau _\text {inj})$. We observe that the curve in momentum space of a classical FEL covers multiples of the recoil $q$ and thus is broadened in comparison to its quantum counterpart which is sharply peaked around $p=q/2$. Moreover, the statistics, $P_n$ against $n$, for a Quantum FEL is narrower as in the classical case which is even more pronounced in the picture on the very right. Here $P_n$ and $f_\text {cl}$ for their respective maximum, at $p=q/2$ and $2kpT/m=\pi$, are compared to a Poisson statistics. Both curves for a small-signal FEL show a super-Poissonian behavior, but due to the additional broadening, Eq. (5), of $f_\text {cl}$ with $1/(\omega _\text {r}T)\gg 1$ the photon statistics of a Quantum FEL is closer to the Poisson distribution.

In the classical regime the small-signal limit is characterized by the Madey gain^27,57^

4 $$\begin{aligned} \mathcal {G}^\text {(cl)}\cong \frac{16}{\pi ^3} \, \omega _\text {r}T (gT)^2 N=\frac{16}{\pi ^3} \omega _\text {r}T \, \mathcal {G} \end{aligned}$$

for the ‘classical resonance’ $2kpT/m \cong \pi$ which gives the maximum of a smooth gain curve and thus differs from the sharp peak at $p=q/2$ for a Quantum FEL. As a consequence, the Fano factor in the classical regime is given by^54,56,58^

5 $$\begin{aligned} \sigma _\text {cl}^2 \cong \frac{\pi /4}{\omega _\text {r}T}\frac{1}{\delta ^{\text {(cl)}}} \end{aligned}$$

which can be for example derived^56^ with an approach relying on a Fokker-Planck equation^55,59,60^. Since Eq. (5) implies the classical limit of a small recoil, that is $\omega _\text {r}T\ll 1$, the Fano factor for the classical FEL is much larger than the corresponding value for the Quantum FEL given by Eq. (3). In other words, the photon statistics of the Quantum FEL in the small-signal regime is in principle closer to a Poissonian than the statistics of the classical FEL, as illustrated in Figure 2.

**Fig. 3:**
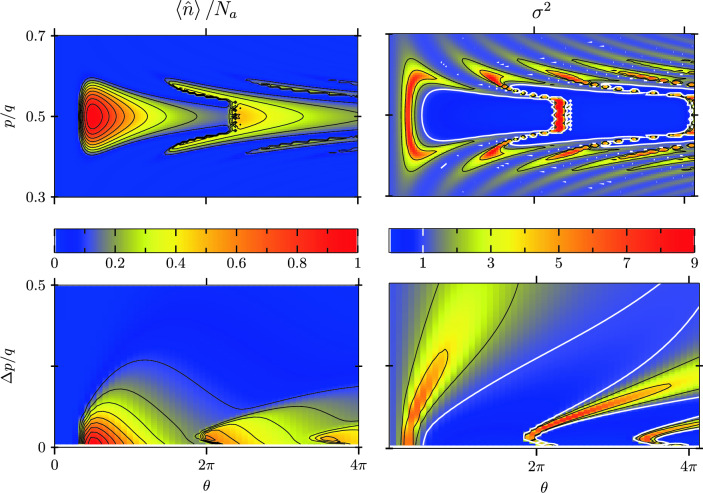
Mean photon number $\langle {\hat{n}}\rangle$ divided by the inverse loss parameter $N_a$ (left) and Fano factor $\sigma ^2\equiv \text {Var}(\hat{n})/\langle {\hat{n}}\rangle$ (right) of a Quantum FEL oscillator for $N_a=150$, and $\alpha _{N_a}=0.1$. Top: as functions of the pump parameter $\theta$ and the momentum $p$ for an initially peaked momentum distribution $\rho (p')=\updelta (p'-p)$ of the electrons. Bottom: as functions of $\theta$ and the standard deviation $\Delta p$ of a Gaussian momentum distribution $\rho (p')=(\sqrt{2\pi }\Delta p)^{-1}\exp [{-(p'-q/2)^2/(2\Delta p^2)}]$ centered at $p=q/2$. For momenta $p$ deviating from resonance and increasing values of $\Delta p$, respectively, the mean photon number $\langle {\hat{n}}\rangle$ decreases. However, this decrease is more slowly for higher values of the pump parameter $\theta$ since there the interaction between electron and field is stronger. On the right-hand side, we observe situations with a sub-Poissonian statistics, that is $\sigma ^2<1$, which is illustrated by the white contour line at $\sigma ^{2}=1$.

In Figure 3 we present the behavior of the mean value $\langle {\hat{n}}\rangle$ and the Fano factor $\sigma ^2$ from the photon statistics $P_n$ of a Quantum FEL oscillator given by Eq. (2) also beyond the small-signal limit. Only momenta close to resonance $p=q/2$ or small momentum widths $\Delta p$ lead to a nonzero mean photon number. In atomic scattering this resonance width is known as velocity-selectivity^61–63^ and solely caused by a Doppler detuning of the momentum distribution. For an efficient operation we even have to go beyond the condition $\Delta p < q$ for the momentum spread $\Delta p$ and demand for

6 $$\begin{aligned} \Delta p < \alpha _n q \ll q \,, \end{aligned}$$

where we have estimated the width of the resonance in momentum space by means of Eq. (1). However, increasing the pump parameter $\theta$ leads to a stronger interaction between electrons and fields and at some point we also observe amplification for off-resonant momenta.

Another feature analogous to the micromaser^45^ is the possibility of a sub-Poissonian photon statistics. Indeed, the Fano factor takes on values which are smaller than unity, that is $\sigma ^2<1$, which is highlighted in Figure 3 by the white contour line for $\sigma ^2=1$. We observe that this sub-Poisson behavior at resonance $p=q/2$ is not fully destroyed by an increasing momentum spread $\Delta p$. Moreover, we expect that also beyond the small-signal limit the photon distribution in the classical regime is very broad^54^. Hence, we identify the sub-Poissonian photon statistics in the quantum regime as a unique feature of a Quantum FEL.

### Experimental challenges

In the following, we discuss the challenges for a possible experimental realization of a Quantum FEL oscillator. For this purpose, we restrict ourselves again to the small-signal limit, that is $gT\sqrt{n} \ll 1$. The coupling constant^56^

7 $$\begin{aligned} g\equiv \frac{e^2 \mathcal {A}_\text {L}\tilde{\mathcal {A}}_\text {W}}{\hbar m}= \frac{a_0}{2\gamma _0} \sqrt{\frac{r_\text {e}c\lambda _\text {W}}{\lambda _\text {C}\sigma _\text {e}^2\tau _\text {e} }} \end{aligned}$$

depends on the mass *m* of an electron, on the amplitude $\tilde{\mathcal {A}}_\text {W}$ of the vector potential for the undulator field and on the vacuum amplitude $\mathcal {A}_\text {L}$ for the laser field. We have rewritten *g* in the second expression in terms of the undulator wavelength $\lambda _\text {W}$, the undulator parameter $a_0$, the electron energy $\gamma _0$, the bunch radius $\sigma _\text {e}$, the bunch duration $\tau _\text {e}$, the classical electron radius $r_\text {e}$, and the Compton wavelength $\lambda _\text {C}$ of an electron. Taking time dilation into account gives the interaction time $T=L(1+a_0^2)^{1/2}/(\gamma _0 c)$ in the Bambini–Renieri frame in terms of the interaction length *L* in the laboratory frame.

A large recoil can be achieved by a small wiggler wavelength $\lambda _\text {W}$ or a high electron energy $\gamma _0$ which becomes more apparent when we write the recoil parameter $\omega _\text {r}T$ in terms of the laboratory frame yielding^56^

8 $$\begin{aligned} 1\ll \omega _\text {r}T \propto \gamma _0 \frac{L}{\lambda _\text {W}}\,. \end{aligned}$$

On the other hand, we demand for a moderately high gain to observe amplification of the laser field. For a given value of the linear gain $\mathcal {G}$ we thus obtain for resonance, $p=q/2$, the relation

9 $$\begin{aligned} L=\sqrt{\mathcal {G}}\frac{c}{g\sqrt{N}}\propto \frac{\gamma _0^2}{\sqrt{\lambda _\text {W}n_\text {e}}} \end{aligned}$$

for the length of the wiggler with $n_\text {e}$ denoting the electron density. For increasing values of $\gamma _0$ the wiggler length *L* grows quadratically while it only scales with $\lambda _\text {W}^{-1/2}$ with the wiggler wavelength. Hence, for simultaneously being in the quantum regime and reducing the length of the wiggler we propose to operate a Quantum FEL oscillator with a moderately high electron energy while decreasing the wiggler wavelength. This need for a small wavelength quite naturally forces us to employ an optical undulator^64–67^ which has also been proposed previously^6^.

**Table 1:** Proposed fundamental parameters for a Quantum FEL oscillator experiment in the small-signal regime.

**Experimental parameters**					
Laser wavelength	$\lambda _\text {L}$ (Å)	1.0	Gain bandwidth	$\Gamma$	$7.45 \cdot 10^{-5}$
Undulator wavelength	$\lambda _\text {W}$ ($\upmu \mathrm{m}$)	1.064	Electron energy	$\gamma _0$	51.8
Recoil parameter	$\omega _\text {r}T$	$2\pi$	Undulator length	*L* (mm)	1.14
Spontaneous emission	$R_\text {sp}L$	0.145	Undulator parameter	$a_0$	0.0944
Space charge	$k_\text {p}L$	0.145	Electron density	$n_\text {e} (\upmu \hbox {m}^{-3})$	$6.39\cdot 10^{4}$
Linear gain	$\mathcal {G}$	0.1			

We have to ensure^12^ that apart from multiphoton effects the impact of (i) space charge and of (ii) spontaneous emission can be neglected. Hence, we additionally require that $k_\text {p}L\ll 1$ as well as $R_\text {sp}L\ll 1$ where we have introduced the plasma wavenumber $k_\text {p}\propto \sqrt{n_\text {e}/\gamma _0^3}$ and the spontaneous decay rate $R_\text {sp}\propto a_0^2/\lambda _\text {W}$ with $a_0$ denoting the undulator parameter. However, these quantities are not independent from each other, but are connected

10 $$\begin{aligned} (k_\text {p}L)^2\cdot (R_\text {sp}L)=\frac{2\alpha _\text {f}}{3}\, \mathcal {G}\cdot (\omega _\text {r}T) \end{aligned}$$

via the gain $\mathcal {G}$ and the fine structure constant $\alpha _\text {f}\approx 1/137$. In contrast to a single-pass FEL^12^, we straightforwardly identify a regime, where all conditions can be simultaneously satisfied. If we for example demand for a gain $\mathcal {G}$ of 10% and set the recoil parameter to $\omega _\text {r}T=2\pi$, Eq. (10) allows for $k_\text {p}L=R_\text {sp}L\approx 0.145$. This more advantageous behavior is due to the low gain which comes hand in hand with a decreased interaction length. We still have the freedom to adjust two parameters to fully determine the set of fundamental parameters $\gamma _0$, $n_\text {e}$, $\lambda _\text {W}$, $a_0$, and $\lambda _\text {L}$. From the choice $\lambda _\text {L}=1\,$Å and $\lambda _\text {W}=1.064\,$µm we derive the values that are listed on the right-hand side of Table 1 (for more details, see the Supplementary Information).

**Fig. 4:**
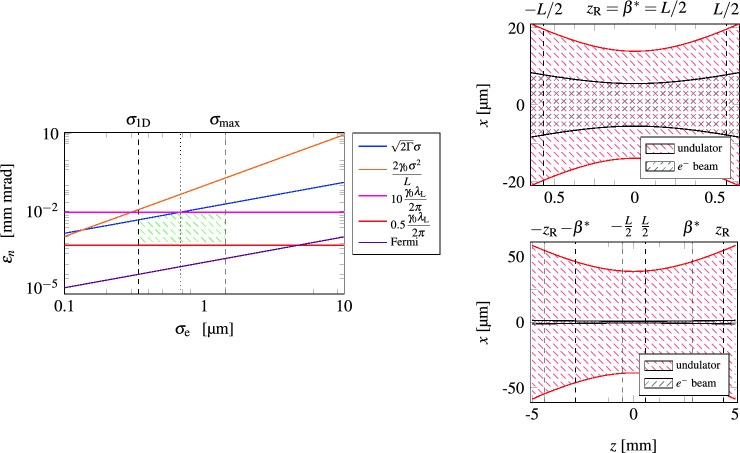
Experimental challenges for a Quantum FEL oscillator: On the left-hand side we present different constraints on the transverse normalized emittance $\epsilon _n$ of the electron beam as functions of the beam radius $\sigma _\text {e}$. Only parameter pairs within the green shaded area fulfill these conditions leading to a situation with $\epsilon _n\sim 10^{-2}\,$mm mrad and $\sigma _\text {e}\sim \,$µm, which is extremely challenging for an experimental realization. On the right-hand side we have shown the longitudinal, *z*, and transversal, *x*, dimensions of the undulator and of the electron beam, when (i) only geometrical considerations are taken into account and when (ii) all conditions are satisfied. While in the former case, both, the Rayleigh length $z_\text {R}$ of the optical undulator and its counterpart $\beta ^*$ for the electron beam coincide with half of the interaction length *L*, that is $z_\text {R}=\beta ^*=L/2$, we observe in the latter case an increased value of $z_\text {R}$ and the relation $L/2< \beta ^* < z_\text {R}$. Hence, the electron beam occupies only a small area of the undulator beam.

Besides such fundamental constraints we have to take geometrical considerations into account. We assume that the optical undulator can be described as a Gaussian laser beam with waist $w_0$ and Rayleigh length $z_\text {R}\equiv \pi w_0^2/\lambda _\text {W}$. Following other studies^12^ we require $w_0\ge \sqrt{2\pi } \sigma _\text {e}$, where $\sigma _\text {e}$ denotes the radius of the electron beam. Moreover, the Rayleigh length $z_\text {R}$ has to be at least as long as half of the interaction length, that is $2z_\text {R}\ge L$. Similar to the Rayleigh length of a light beam, the quantity $\beta ^*\equiv \sigma _\text {e}^2\gamma _0 /\epsilon _n$ describes the typical length for the divergence of an electron beam and the normalized transverse emittance $\epsilon _n$ is connected to the area of the beam in phase space^46^. From $2\beta ^*\ge L$ we derive^12^ the condition $\epsilon _n\le \sigma _\text {e}^2\gamma _0/L$.

We also have to relate the dimensions of the electron beam to the emitted radiation^12^ characterized by the beam emittance $\lambda _\text {L}/(4\pi )$. A compromise between a strongly diverging laser beam ($\lambda _\text {L}/(4\pi )> \epsilon _n/\gamma _0$) and the loss of transverse coherence ($\lambda _\text {L}/(4\pi )\ll \epsilon _n/\gamma _0$) is typically found in the regime^12^

11 $$\begin{aligned} 0.5\, \frac{\gamma _0\lambda _\text {L}}{2\pi } < \epsilon _n \le 10\, \frac{\gamma _0\lambda _\text {L}}{2\pi }\,. \end{aligned}$$

Indeed, this relation that ensures transverse coherence was originally derived^68,69^ for self-amplified spontaneous emission (SASE). However, the radiation in an oscillator also starts up from vacuum and reaches steady state after passages of many electron bunches. There is no mechanism evident inducing transverse coherence and we consequently demand that Eq. (11) is also satisfied for an oscillator, assuming transverse coherence for the start-up from vacuum.

In Table 1 we have also included the value for the gain bandwidth $\Gamma$. In the small-signal limit the condition from momentum selectivity, Eq. (6), reads $\Delta p \le q/(\omega _\text {r}T)$ and we obtain

12 $$\begin{aligned} \frac{\Delta \gamma _0}{\gamma _0}\le \Gamma \equiv \frac{\lambda _\text {W}}{4\pi L} \end{aligned}$$

which results in a challenging value for the electron energy spread at the order of $\sim 10^{-4}$. We can relate fluctuations and deviations of several parameters to the gain bandwidth $\Gamma$. For this purpose, we note the fundamental relation^12,70^

13 $$\begin{aligned} \lambda _\text {L}=\frac{\lambda _W}{2\left( 1-\cos {\phi }\right) \gamma _0^2} \left( 1+a_0^2+\gamma _0^2\vartheta ^2\right) \,, \end{aligned}$$

where $\vartheta$ is the emission angle and $\phi$ denotes the angle between the propagation directions of electrons and optical undulator. For example, we find the conditions $\Delta \lambda _\text {W}/\lambda _\text {W}\le 2\Gamma$ and $\Delta I_0/I_0\le 2\Gamma /a_0^2$ for the bandwidth and the intensity fluctuations of the undulator laser with intensity $I_0\propto a_0^2$. Divergence of the electron beam leads to deviations from the head-on geometry ($\phi =\pi$ and $\vartheta =0$) resulting in the condition^12^ $\varepsilon _n\le \sigma _\text {e} \sqrt{2\Gamma }$, where we have set $\gamma _0 \epsilon _n = \updelta \phi \sigma _\text {e}$.

Moreover, one can derive requirements for the available interaction area due to longitudinal and transversal intensity variations of the undulator laser close to the focus, resulting in $\Delta z/ z_\text {R}\le \sqrt{2\Gamma /a_0^2}$, and, $\Delta x/w_0 \le \sqrt{\Gamma /a_0^2}$, respectively^12^. For a minimized beam waist we thus derive a maximum radius of $\sigma _\text {max}=[\Gamma /(2a_0^2)]^{1/4}\sqrt{\lambda _\text {W}L}/(2\pi )$ for the electron beam.

For the sake of completeness, we have to exclude Fermionic effects by demanding^5^ that the number *N* of electrons is smaller than the phase space volume of the electron bunch divided by $\hbar ^3$ leading to the condition

14 $$\begin{aligned} \epsilon _n> \sigma _\text {e} \sqrt{\left( \frac{\lambda _\text {C}}{2\pi }\right) ^3\frac{\pi \gamma _0 n_\text {e}}{\Gamma }}\,. \end{aligned}$$

We also have to ensure that our one-dimensional approach is correct and require^51^ $\sigma _\text {e}> \sigma _{1\text {D}}\equiv \sqrt{L \lambda _\text {L}}$.

We visualize the conditions for the radius $\sigma _\text {e}$ and the normalized emittance $\epsilon _n$ of the electron beam on the left-hand side of Fig. 4 at hand of the parameters in Table 1. The regime which allows for a realization of a Quantum FEL oscillator is indicated by a green shading. We realize that the required beam radius lies in the µm range with a normalized emittance at the order of $10^{-2}\,$mm mrad, which are extremely challenging requirements for an experiment. On the right-hand side of Fig. 4 we sketch the longitudinal and transverse dimensions of electron beam and undulator laser, when (i) only the geometrical requirements are met, that is $z_\text {R}=\beta ^*=L/2$ (top), and (ii) all conditions are satisfied leading to $z_\text {R}>\beta ^*>L/2$ (bottom). We observe that $z_\text {R}$ and $w_0$ increase in the latter case, but at the same time the electron beam occupies only a small area of the undulator beam. To improve the situation, a traveling-wave Thomson-scattering scheme (TWTS) has been proposed^12,67^, where the undulator laser has a tilted front and copropagates with the electron beam under an certain angle. While this method relaxes the conditions for the optical undulator, it has no influence on the strict requirements for the electron-beam quality.

**Table 2:** Proposed parameters for the electron beam and the optical undulator of a low gain Quantum FEL oscillator for the target parameters $\omega _\text {r}T=2\pi$, $\mathcal {G}=0.1$, $R_\text {sp}L=k_\text {p}L=0.145$, and $L=1.1$ mm listed in Table 1. The values are chosen such that $10^6$ photons per pulse are coupled out of a cavity with reflectivity $R=95.05$ % at the wavelength $\lambda _\text {L}=1$ Å. The FEL operation reaches steady state at about $2\cdot 10^4$ round trips.

**Electron beam**			**Optical undulator**		
Electron energy	$\gamma _0$	52	Undulator wavelength	$\lambda _\text {W}$ ($\upmu \text {m}$)	1.064
Electron density	$n_\text {e}$ ($\upmu \text {m}^{-3}$)	$6.4\cdot 10^{4}$	Undulator parameter	$a_0$	0.094
Bunch radius	$\sigma _\text {e}$ ($\upmu \text {m}$)	0.68	Beam waist	$w_0$ ($\upmu \text {m}$)	38.6
Trans. norm. emittance	$\epsilon _n$ (mm mrad)	0.0082	Rayleigh length	$z_\text {R}$ (mm)	4.4
Beam duration	$\tau _\text {e}$ (ps)	1.2	Pulse duration	$\tau _0$ (ps)	7.6
Peak current	$I_\text {p}$ (A)	8.9	Intensity	$I_0$ $\left(\frac{\text {PW}}{\text {cm}^2} \right)$	21.7
Bunch charge	*Q* (pC)	11	Laser power	$P_0$ (TW)	0.5
Electron number (bunch)	*N*	$6.9 \cdot 10^7$	Undulator linewidth	$\displaystyle \frac{\Delta \lambda _\text {W}}{\lambda _\text {W}}$ (‰)	0.15
Energy spread	$\displaystyle \frac{\Delta \gamma _0}{\gamma _0}$ (‰)	0.075	Intensity fluctuations	$\displaystyle \frac{\Delta I_0}{I_0}$ (%)	1.7

Similar to the Rayleigh length of the undulator, its pulse duration $\tau _0$ has to be larger than twice the interaction length divided by *c*, that is $\tau _0>2L/c\sim 10\,$ps. Further, neglecting slippage implies^51^ that the bunch length $c\tau _\text {e}$ is larger than the number $L/\lambda _W$ of undulator periods times the emitted wavelength $\lambda _\text {L}$ which means for our case that the beam duration $\tau _\text {e}$ has to be larger than approximately $0.3\,$fs. The beam duration is the missing parameter to determine the bunch charge and with that the number of emitted photons. For XFELOs a gain reduction is expected when the beam duration is at the order of the inverse bandwidth of the cavity crystals that is typically in the sub-picosecond regime^20^. We therefore require a beam duration larger than 1 ps. A set of possible parameters for electron beam and optical undulator of a Quantum FEL oscillator is listed in Table 2. We note that fluctuations of the electron number from bunch to bunch have to be small enough such that the gain $\mathcal {G}\propto N$ is always larger than the cavity losses and thus we require $\Delta n_\text {e}/n_\text {e}\ll \delta$ for the density fluctuations $\Delta n_\text {e}/n_\text {e}$ compared to the relative deviation $\delta$ of the gain from threshold.

A detailed study of the x-ray cavity lies outside the scope of the present article. Instead, we briefly summarize in the following the most important relations and conditions: The back and forward reflected FEL pulses in the resonator have to arrive at the same time as the electron bunches at the interaction area. Hence, we require $L_\text {cav}=c/(2f_\text {rep})$ for the cavity length, where we have introduced the repetition rate $f_\text {rep}=1/\tau _\text {inj}$ of the accelerator. In experimental implementations this rate is limited and consequently the cavity becomes large. For example, a very optimistic repetition rate of 10 MHz implies a cavity length of 15 m. Despite the small dimensions of the optical undulator, a Quantum FEL oscillator would thus be far away from being a compact device.

The steady-photon number $n_\text {st}\equiv \langle {\hat{n}}\rangle$ can be determined via the relation $n_\text {st}=3\delta \, N/\mathcal {G}$. Further, the quality of the resonator is fundamentally tied to its reflectivity *R* and its length^60^ $L_\text {cav}$ and with $L_\text {cav}=c/(2f_\text {rep})$ we find the relation

15 $$\begin{aligned} R=1-\frac{\omega _\text {L}\tau _\text {inj}}{2Q}\,. \end{aligned}$$

A relative deviation $\delta$ of gain from losses of 1% (corresponding to $gT\sqrt{n_\text {st}}\approx 0.17$) thus translates to a reflectivity of 95.05%. We assume that there are no losses due to absorption so that 4.95 % of the photons are transmitted through a thin mirror. For our set of parameters this ratio translates to $10^6$ outcoupled photons per pulse. Before saturating and reaching steady state, the laser field builds up from vacuum. We estimate the number of repetitions $N_\text {rep}$ of electron bunches for reaching steady state via the relation $N_\text {rep}\cong \log {(3 N \delta /\mathcal {G}^2)}/( \mathcal {G} \delta )$ (see Supplementary Information for details) and find $N_\text {rep}\sim 2\cdot 10^4$ for the parameters from Table 2. Regarding the temporal structure of each individual pulse, we expect a similar behavior like a classical FEL oscillator. The proposed XFELO emits single-peak pulses whose duration is determined by the electron-bunch duration^20^ $\tau _\text {e}$, which is on the order of ps in our example. Whether the finite pulse duration $\tau _0$ of the optical-undulator pulse, which is only one order of magnitude larger than $\tau _\text {e}$, affects this behavior requires a more detailed investigation, which lies beyond the scope of the present work.

We note that focusing the cavity mode to the size of the electron beam at its waist $\sim$µm would lead to a Rayleigh length in the cm-regime which is well above the interaction length. To ensure that the cavity is in resonance with the FEL mode we further require that fluctuations in the cavity length $L_\text {cav}$ due to vibrations or analogously effective length changes from timing errors due to fluctuations in the accelerator^19^ are small. By means of Eq. (13) we derive $\Delta L_\text {cav}/L_\text {cav}\le 2\Gamma$ which translates to a length change in the mm to cm regime depending on the cavity length. However, the timing error has to be also much smaller than the inverse bandwidth of the cavity which typically leads to a maximum length change in the µm regime^19^. Moreover, the x rays produce a heat load on the crystals affecting their reflectivity so that a cooling of the mirrors to a temperature below 100 K becomes necessary^71^.

The exact timing of electron bunches, cavity field, and undulator is essential for the operation of a Quantum FEL oscillator, especially since we replace the usual magnetostatic undulator with a pulsed high-power laser. Generating laser pulses with $\sim$ TW peak power and pulse duration $\sim 10$ ps with a repetition rate that matches the rate of electron bunches at $\sim 10$ MHz are beyond the capabilities of current laser systems, which operate in the best scenarios with rates in the kHz-regime for similar pulse parameters^72–74^. Reducing the repetition rate of electron bunches provides only a modest improvement: A reduction by one order of magnitude to $f_\text {rep}=1$ MHz implies a cavity length of 150 m, and a further decrease to 10 kHz would lead to unrealistic value of $L_\text {cav}=15$ km. However, as apparent in Fig. 4, a large part of the cross section of the optical undulator does not contribute to the interaction with the electrons for a head-on geometry. TWTS schemes^12,67^ increase the efficiency of the interaction and therefore constitute a possible route to lower the required laser power, bringing the specifications for an optical undulator closer to realistic values. Similarly, it is challenging to provide high-quality electron beams with sufficient repetition rates. For example, plasma wakefield acceleration could produce electron bunches with low emittance and low energy spread^49^, but plasma damping introduces a recovery time that limits the attainable repetition rate^50^. While at least in principle the upper limit for this rate^50^ is at roughly 10 MHz, that is, of the order required for our parameters, reaching this limit in practice remains challenging.

## Discussion

The steady-state photon statistics of a Quantum FEL oscillator is closer to the Poisson distribution of a coherent state and eventually shows a sub-Poissonian behavior. Beside the benefit of demonstrating this pure quantum effect, a narrowed photon statistics would lead to smaller intensity fluctuations. In imaging applications, this reduction implies for a fixed value of $\langle \hat{n} \rangle$ a smaller signal-to-noise ratio, which is connected to the inverse Fano factor. Besides intensity fluctuations limiting imaging, the sensitivity of interferometric applications depends on the photon statistics of the interference pattern and therefore benefits from a narrower photon distribution.

The interaction length of a low-gain FEL oscillator is smaller compared to a single-pass device. As a result, the conditions on multiphoton effects, spontaneous emission, and space charge can be simultaneously fulfilled. Although the shorter interaction length also improves the challenges for an optical undulator, the limits due to focusing are still prominent, at least for head-on geometries. However, TWTS schemes^67^ could help to overcome this issue. Unfortunately, a Quantum FEL oscillator can be hardly reduced in size to be a compact device, since the cavity is much longer than the interaction length due to a limited electron bunch repetition rate. For the quality of the electron beam there are very strict criteria (also because the gain bandwidth for a Quantum FEL oscillator is very small). At least, the shorter interaction length might reduce the effect that the macroscopic beam dynamics^12^ impairs the beam quality along the undulator.

Already proposed schemes of Quantum FEL operation rely either on SASE^2^ or a cascade of Quantum FELs, where each output serves as seed for the next FEL^12^. The latter setup is similar to the concept of an oscillator without a resonator that stabilizes the FEL at steady state. Moreover, the proposed oscillator can operate in the low-gain regime in contrast to the seeded high-gain FEL, which implies the mentioned differences in the scaling of the interaction length.

Classical FEL oscillators play an important role for longer wavelengths, for example in the THz regime^75–77^. The basic scheme of amplifying undulator radiation in many round trips in a resonator is very similar for all wavelengths, and in all cases the timing condition for electron bunches and the amplified radiation implies that the cavity length is determined by the repetition rate of the electron accelerator. However, THz devices have much weaker requirements for energy and quality of the electron beam^75^ than x-ray FELs or even Quantum FELs. Moreover, high-quality mirrors for THz radiation are state-of-the-art components compared to the Bragg mirrors in the x-ray regime, which are in a much earlier stage of design. Substituting magnetostatic undulators with pulsed high-power lasers creates new experimental challenges, especially with respect to the exact timing of electron bunches, the undulator laser pulses, and the FEL mode.

Indeed, key specifications of individual components seem achievable with current or near-future technology. However, the detailed interplay of all constraints makes the simultaneous the fulfillment of all requirements purely prospective, especially the need for a high repetition rate of high-power laser pulses and for a matching rate of high-quality electron bunches.

## Supplementary Information

Supplementary Information.

## Data Availability

The datasets used and/or analysed during the current study are available from the corresponding author on reasonable request.
