# Supplementary material for: Quantum free-electron laser oscillator
Source: Sci Rep. 2026 Mar 30;16:10521. doi: 10.1038/s41598-026-45068-1 (PMC13035920; doi:10.1038/s41598-026-45068-1)
Supplement: Supplementary file 1 — Supplementary Information. [file 41598_2026_45068_MOESM1_ESM.pdf]

Quantum free-electron laser oscillator: Supplementary Information

Peter Kling[1,*] **and Enno Giese**[2]

[1]German Aerospace Center (DLR), Institute of Quantum Technologies, Ulm, 89081, Germany
[2]Technische Universität Darmstadt, Fachbereich Physik, Institut für Angewandte Physik, Darmstadt, 64289, Germany

ABSTRACT

This supplementary information summarizes key steps in the derivation of the photon statistics of a Quantum FEL oscillator and experimental parameters for the operation of this device.

S1 Photon statistics

S1.1 Density operator approach

The photon statistics P_n is given by the diagonal elements

$$P_n(t) \equiv \langle n| \hat{\rho}_{\mathrm{L}}(t) |n\rangle = \langle n| \mathrm{Tr}_{\mathrm{el}} \{\hat{\rho}(t)\} |n\rangle \tag{S.1}$$

of the reduced density operator for the radiation field in photon-number representation, with $\hat{\rho} = \hat{\rho}(t)$ denoting the density operator for the combined system of electron and radiation field. The equation of motion of this photon number probability

$$\dot{P}_n(t) = \left(\frac{\partial P_n(t)}{\partial t}\right)_{\mathrm{int}} + \left(\frac{\partial P_n(t)}{\partial t}\right)_{\mathrm{loss}} \tag{S.2}$$

can be divided into two parts, that is (i) the interaction of the electrons with the undulator and the radiation field and (ii) cavity losses.

Let us first consider the interaction part: At a time t prior to interaction the density operator of the total system is given by the tensor product of (i) the electronic part $|\Phi_{\mathrm{el}}\rangle \langle\Phi_{\mathrm{el}}|$ with the state vector

$$|\Phi_{\mathrm{el}}\rangle = \int \mathrm{d}p\, \phi(p) \,|p\rangle \tag{S.3}$$

being described by the wave function $\phi = \phi(p)$ in momentum representation, and (ii) the density operator $\hat{\rho}_{\mathrm{L}} = \hat{\rho}_{\mathrm{L}}(t)$ for the laser field. During the interaction time T, the state evolves from $\hat{\rho}(t)$ to $\hat{\rho}(t+T)$. This evolution is given by Rabi oscillations[1] between the two momentum levels p and $p-q$, where one photon is emitted into the field or re-absorbed by the electron with the momentum dependent Rabi frequency

$$\Omega_n(p) \equiv \sqrt{g^2(n+1) + \omega_{\mathrm{r}}^2 \left(\frac{p}{q} - \frac{1}{2}\right)^2}\,. \tag{S.4}$$

Here we have introduced the coupling constant g, the quantum mechanical recoil $q \equiv 2\hbar k$, and the recoil frequency $\omega_{\mathrm{r}} \equiv q^2/(2m\hbar)$. The second term of the square root accounts for a Doppler detuning across the momentum distribution of the electrons. In terms of momentum eigenstates $|p\rangle$ and photon-number states $|n\rangle$ this dynamics translates to the substitution

$$|n,p\rangle \to c_n(p,T)\,|n,p\rangle + c_{n+1}(p-q,T)\,|n+1,p-q\rangle \tag{S.5}$$

with the coefficients

$$c_n(p,T) = \cos\left[\Omega_n(p)T\right] - i\frac{\omega_{\mathrm{r}}}{\Omega_n(p)}\left(\frac{p}{q} - \frac{1}{2}\right)\sin\left[\Omega_n(p)T\right] \tag{S.6}$$

and

$$c_{n+1}(p-q,T) = -i\,\frac{g\sqrt{n+1}}{\Omega_n(p)}\sin\left[\Omega_n(p)T\right]. \tag{S.7}$$

A straightforward calculation leads to the expression

$$P_n(t+T)-P_n(t)=-\frac{n+1}{N}\,G_n\,P_n(t)+\frac{n}{N}\,G_{n-1}\,P_{n-1}(t) \tag{S.8}$$

for the change of P_n during the interaction, where we have defined

$$G_n \equiv (gT)^2 N \int \mathrm{d}p\,\mathrm{sinc}^2\left[\Omega_n(p)T\right]\rho(p), \tag{S.9}$$

which corresponds to the non-linear gain of the laser field with the definition of the electrons' momentum distribution $\rho(p)\equiv|\phi(p)|^2$. For the change of P_n due to the interaction of the field with the whole electron bunch we multiply the result in Eq. (S.8) with the number N of electrons in a bunch.

In the oscillator configuration many electron bunches are subsequently injected with a rate $f_{\mathrm{rep}}=1/\tau_{\mathrm{inj}}$. We approximate with the help of the coarse-grained derivative[2]

$$\left(\frac{\partial P_n(t)}{\partial t}\right)_{\mathrm{int}} \cong \frac{N}{\tau_{\mathrm{inj}}}\left[P_n(t+T)-P_n(t)\right] \tag{S.10}$$

the continuous change of P_n with a discrete one. The contribution to the dynamics of P_n due to cavity losses reads

$$\left(\frac{\partial P_n(t)}{\partial t}\right)_{\mathrm{loss}} = \frac{\omega_{\mathrm{L}}}{Q}\left[(n+1)\,P_{n+1}(t)-n\,P_n(t)\right], \tag{S.11}$$

with Q denoting the quality factor of the cavity. This result can be derived within a Born–Markov approach[3,4]. In the x-ray regime of the spectrum we neglect the number of thermal photons in the laser mode, that is $n_{\mathrm{th}}=0$. Finally, we obtain the equation of motion

$$\dot{P}_n(t)=-(n+1)\left[\frac{G_n}{\tau_{\mathrm{inj}}}P_n(t)-\frac{\omega_{\mathrm{L}}}{Q}P_{n+1}(t)\right]+n\left[\frac{G_{n-1}}{\tau_{\mathrm{inj}}}P_{n-1}(t)-\frac{\omega_{\mathrm{L}}}{Q}P_n(t)\right] \tag{S.12}$$

for the photon statistics of the Quantum FEL oscillator. For steady state, that is $\dot{P}_n=0$, it suffices that the expressions in square brackets vanish separately[3,4]. With the help of this detailed-balance condition we derive the formal solution

$$\frac{P_n}{P_0}=\prod_{n'=1}^{n}\frac{G_{n'-1}}{\omega_{\mathrm{L}}\tau_{\mathrm{inj}}/Q}\equiv\prod_{n'=1}^{n}\Lambda_{n'} \tag{S.13}$$

for the steady-state photon statistics that corresponds to Eq. (2) in the main body of this article.

S1.2 Gaussian approximation of photon statistics

To analyze the properties of the photon statistics a closed, analytic solution is useful. Hence, we perform in the following a Gaussian approximation[5,6] of the photon statistics. Therefore, we approximate the discrete sum in Eq. (S.13) as an integral, that is

$$\frac{P_n}{P_0}=\exp\left(\sum_{n'=1}^{n}\ln\Lambda_{n'}\right)\cong \mathrm{e}^{\mathscr{I}(n)}, \tag{S.14}$$

where we have defined

$$\mathscr{I}(n)\equiv\int_1^n \mathrm{d}n'\,\ln\Lambda(n'). \tag{S.15}$$

as a continuous function of n.

We determine the maximum of this distribution at $n\equiv n_{\mathrm{st}}$ by setting $(\mathrm{d}P_n/\mathrm{d}n)_{n=n_{\mathrm{st}}}=0$, which translates to the necessary condition $\Lambda(n_{\mathrm{st}})=1$. Moreover, we require $(\mathrm{d}\Lambda(n)/\mathrm{d}n)_{n=n_{\mathrm{st}}}<0$ to ensure that n_{st} really describes a maximum of P_n. In the next step we expand

$$\mathscr{I}(n)\cong\mathscr{I}(n_{\mathrm{st}})+\frac{1}{2}\left.\frac{\mathrm{d}\Lambda(n)}{\mathrm{d}n}\right|_{n=n_{\mathrm{st}}}(n-n_{\mathrm{st}})^2 \tag{S.16}$$

around $n = n_{\rm st}$. This expansion leads to the result

$$P(n) \cong \frac{1}{\sqrt{2\pi\Delta n^2}} \exp\left[-\frac{(n-n_{\rm st})^2}{2\Delta n^2}\right] \tag{S.17}$$

for the steady-state photon statistics, which corresponds to a Gaussian with the mean value $n_{\rm st}$ defined by $\Lambda(n_{\rm st}) = 1$, and the variance

$$\Delta n^2 \equiv -\left(\frac{{\rm d}\Lambda(n)}{{\rm d}n}\bigg|_{n=n_{\rm st}}\right)^{-1}. \tag{S.18}$$

For exact resonance, $\rho(p) = \delta(p - q/2)$, we recover analog results as for the micromaser[5], that is, (i) the transcendental equation

$$\frac{\omega_{\rm L}\tau_{\rm inj}}{Q}\frac{n_{\rm st}}{N} = \sin^2\left(gT\sqrt{n^{\rm st}}\right) \tag{S.19}$$

for the mean photon number $n_{\rm st} \equiv \langle \hat{n} \rangle$ and the expression

$$\sigma^2 = \frac{1}{1-\left(gT\sqrt{n_{\rm st}}\right)\cot\left(gT\sqrt{n_{\rm st}}\right)} \tag{S.20}$$

for the normalized variance ("Fano factor") $\sigma^2 \equiv \Delta n^2/\langle \hat{n} \rangle$.

S1.3 Small-signal limit

In the following, we restrict ourselves to the small-signal limit. In the quantum regime this limit is defined by the condition $\xi \equiv gT\sqrt{n_{\rm st}} \ll 1$. A Taylor expansion of the right-hand side of Eq. (S.19) in powers of ξ yields the expression $\sin^2\xi \cong \xi^2 - \xi^4/3$ and we straightforwardly arrive at the steady-state photon number $n_{\rm st} = 3N\delta/\mathscr{G}$ in the small-signal limit, where we have defined the linear gain $\mathscr{G} \equiv (gT)^2 N$ as well as the relative deviation $\delta \equiv (\mathscr{G} - \omega_{\rm L}\tau_{\rm inj}/Q)/\mathscr{G}$ from threshold. Analogously, we expand the denominator in Eq. (S.20) in powers of ξ and arrive at $1 - \xi\cot\xi \cong \frac{1}{3}\xi^2$. With the help of the expression for $n_{\rm st}$, we obtain the result $\sigma^2 = 1/\delta$ for the normalized variance in the small-signal limit in terms of the deviation δ from threshold.

We also estimate how many repetitions are necessary for reaching steady state. For this purpose, we derive from Eq. (S.12) the equation of motion

$$\dot{n}(t) \cong \left(\frac{\mathscr{G}}{\tau_{\rm inj}} - \frac{\omega_{\rm L}}{Q}\right) n(t) - \frac{\mathscr{G}^2}{3N} n^2(t) \tag{S.21}$$

for the mean photon number $n(t) \equiv \langle \hat{n}(t) \rangle = \sum_n nP_n(t)$, where we have assumed a sharply peaked photon statistics to neglect higher moments of the photon-number operator[3]. The solution[7]

$$n(t) = \frac{n_0 \exp\left(\frac{\mathscr{G}\delta}{\tau_{\rm inj}} t\right)}{1 - \frac{n_0}{n_{\rm st}}\left[1 - \exp\left(\frac{\mathscr{G}\delta}{\tau_{\rm inj}} t\right)\right]} \tag{S.22}$$

of this differential equation shows that steady state is only asymptotically obtained for $t \to \infty$. For an estimate of the typical time scale, we identify the saturation time $t_{\rm sat}$ as the turning point between the exponential start-up and saturation. Through this procedure we find

$$N_{\rm rep} \cong \frac{1}{\mathscr{G}\delta} \log \frac{3N\delta}{\mathscr{G}^2} \tag{S.23}$$

for the number $N_{\rm rep} = t_{\rm sat}/\tau_{\rm inj}$ of repetitions necessary for saturation, where we have set the initial photon number n_0 equal to the number of spontaneously emitted photons into the laser mode[8], that is $n_0 = (gT)^2 N = \mathscr{G}$.

S2 Experimental parameters

S2.1 Fundamental constraints

Our analysis of the Quantum FEL dynamics[1] is formulated in terms of the co-moving Bambini–Renieri reference frame[9,10]. For experimental considerations, however, it is useful to express the most important quantities of our theory in terms of the laboratory frame, which is done on the left-hand side of Table S1.

Experimental parameters			
laser wavelength	$\lambda_\mathrm{L} = \frac{\lambda_\mathrm{W}}{4\gamma_0^2}(1+a_0^2)$	electron energy	$\gamma_0 = \frac{1}{2}\sqrt{\frac{\lambda_\mathrm{W}}{\lambda_\mathrm{L}}}\left[1+a_0^2(\lambda_\mathrm{L},\lambda_\mathrm{W},\omega_\mathrm{r}T,R_\mathrm{sp}L)\right]$
recoil parameter	$\omega_\mathrm{r}T = 16\pi\gamma_0\frac{L}{1+a_0^2}\frac{\lambda_\mathrm{C}}{\lambda_\mathrm{W}^2}$	undulator length	$L = \omega_\mathrm{r}T\frac{\sqrt{\lambda_\mathrm{W}^3\lambda_\mathrm{L}}}{8\pi\lambda_\mathrm{C}}\left[1+\frac{1}{2}a_0^2(\lambda_\mathrm{L},\lambda_\mathrm{W},\omega_\mathrm{r}T,R_\mathrm{sp}L)\right]$
spontaneous emission	$R_\mathrm{sp}L = \frac{(2\pi)^2}{3}\frac{a_0^2 r_\mathrm{e}}{\lambda_\mathrm{W}\lambda_\mathrm{C}}L$	undulator parameter	$a_0 = \left[\frac{6R_\mathrm{sp}L}{\pi\omega_\mathrm{r}T}\frac{\lambda_\mathrm{C}^2}{r_\mathrm{e}\sqrt{\lambda_\mathrm{W}\lambda_\mathrm{L}}}\right]^{1/2}$
space charge	$k_\mathrm{p}L = \sqrt{\frac{4\pi r_\mathrm{e} n_\mathrm{e}}{\gamma_0^2}}L$	electron density	$n_\mathrm{e} = \frac{2\pi(k_\mathrm{p}L)^2}{(\omega_\mathrm{r}T)^2}\frac{\lambda_\mathrm{C}^2}{r_\mathrm{e}\lambda_\mathrm{W}^{3/2}\lambda_\mathrm{L}^{5/2}}\left[1+\frac{1}{2}a_0^2(\lambda_\mathrm{L},\lambda_\mathrm{W},\omega_\mathrm{r}T,R_\mathrm{sp}L)\right]$
linear gain	$\mathscr{G} = \frac{\pi r_\mathrm{e}\lambda_\mathrm{W} n_\mathrm{e}}{4\gamma_0^2\lambda_\mathrm{C}}L^2 a_0^2(1+a_0^2)$	gain bandwidth	$\Gamma = \frac{\lambda_\mathrm{W}}{4\pi L(\lambda_\mathrm{L},\lambda_\mathrm{W},\omega_\mathrm{r}T,R_\mathrm{sp}L)}$

Table S1. Central parameters for a Quantum FEL oscillator experiment in the small-signal regime.

For the quantum regime, we require $\omega_\mathrm{r}T \gg 1$. The low-gain, small-signal limit is reached when the linear gain $\mathscr{G} = (gT)^2N$ is smaller than unity, that is $\mathscr{G} \ll 1$, and when simultaneously $gT\sqrt{n_\mathrm{st}} \ll 1$. However, $\mathscr{G}$ should not be *too* small, since we need an appreciable amplification exceeding the cavity losses. The coupling constant

$$g\sqrt{N} = \frac{e^2\mathscr{A}_\mathrm{L}\tilde{\mathscr{A}}_\mathrm{W}\sqrt{N}}{\hbar m} = \frac{\sqrt{\pi}a_0 c}{2\gamma_0}\sqrt{\frac{r_\mathrm{e}\lambda_\mathrm{W} n_\mathrm{e}}{\lambda_\mathrm{C}}} \tag{S.24}$$

depends on the amplitude $\tilde{\mathscr{A}}_\mathrm{W}$ of the vector potential for the undulator field and on the vacuum amplitude $\mathscr{A}_\mathrm{L}$ for the laser field. Taking time dilation into account gives the interaction time $T = L(1+a_0^2)^{1/2}/(\gamma_0 c)$ in the Bambini–Renieri frame in terms of the undulator length L in the laboratory frame.

Beside the constraints for recoil and gain, we require that (i) spontaneous emission and (ii) space charge effects can be neglected, that is , $R_\mathrm{sp}L < 1$ and $k_\mathrm{p}L < 1$, respectively. However, these parameters are not independent from each other[11] and we can relate them and the fine-structure constant $\alpha_\mathrm{f} = 2\pi r_\mathrm{e}/\lambda_\mathrm{C}$ via

$$(k_\mathrm{p}L)^2\cdot(R_\mathrm{sp}L) = \frac{2\alpha_\mathrm{f}}{3}\mathscr{G}\cdot(\omega_\mathrm{r}T), \tag{S.25}$$

which can be derived from the expressions in Table S1.

Our strategy to identify parameter regimes where a Quantum FEL operation is possible, starts by fixing the values of $\omega_\mathrm{r}T$, $\mathscr{G}$, $R_\mathrm{sp}L$, $k_\mathrm{p}L$ within the possible ranges, while taking Eq. (S.25) into account. In the main text of this paper we have set $\omega_\mathrm{r}T = 2\pi$, $\mathscr{G} = 0.1$, and solved Eq. (S.25) with the simplifying assumption $R_\mathrm{sp}L = k_\mathrm{p}L$. In the second step, we express the fundamental experimental parameters as functions of the target parameters. Indeed, each set contains five parameters (λ_W, γ_0, L, a_0, n_e) and (λ_L, $\omega_\mathrm{r}T$, $\mathscr{G}$, $R_\mathrm{sp}L$, $k_\mathrm{p}L$). However, because of Eq. (S.25), we can choose only four target parameters independently, leaving us with the freedom of fixing an additional parameter from the other set, for example λ_W. The resulting relations for the fundamental experimental parameters are shown on the right-hand side of Table S1.

For an initial momentum distribution with a nonzero width Δp, the gain is reduced due to velocity selectivity. The lowest order of the expansion of G_{n-1} from Eq. (S.9) in powers of $\xi = gT\sqrt{n}$ gives

$$G_{n-1} \approx \mathscr{G}\int \mathrm{d}p\,\mathrm{sinc}^2\left[\omega_\mathrm{r}T\left(\frac{p}{q}-\frac{1}{2}\right)\right]\rho(p)\,. \tag{S.26}$$

The width of a momentum distribution centered at resonance $q = q/2$ has to be smaller than the width of the sinc^2-function in Eq. (S.26) , that is $\Delta p < q/(\omega_\mathrm{r}T)$. In this case, the overlap in the integral kernel of Eq. (S.26) is large and the gain is not much reduced. In the laboratory frame, this condition translates to $\Delta\gamma_0/\gamma_0 < \Gamma$ for the energy spread $\Delta\gamma_0/\gamma_0$ of the electrons with the gain bandwidth Γ from Table S1.

S2.2 Advanced constraints

We have listed all relevant constraints for an experimental realization of the Quantum FEL oscillator in Table S2.

Experimental constraints	
small-signal limit	$gT\sqrt{n_{\mathrm{st}}} = \sqrt{3\delta} \ll 1$
quantum regime	$\omega_{\mathrm{r}} T \gg 1$
electron energy spread	$\frac{\Delta\gamma_0}{\gamma_0} < \Gamma = \frac{\lambda_{\mathrm{W}}}{4\pi L}$
low gain	$\mathscr{G} = (gT)^2 N \ll 1$
steady-state photon number	$n_{\mathrm{st}} = \frac{3\delta N}{\mathscr{G}}$
spontaneous emission	$R_{\mathrm{sp}} L < 1$
space charge	$k_{\mathrm{p}} L < 1$
undulator parameter[1]	$a_0 = \frac{\sqrt{2} e \tilde{\mathscr{A}}_{\mathrm{W}}}{m_0 c}$
undulator intensity	$I_0 = \frac{\pi m_0 c^3}{r_{\mathrm{e}}} \left(\frac{a_0}{\lambda_{\mathrm{W}}}\right)^2$
undulator laser power	$P_0 = \frac{1}{2} I_0 \pi w_0^2$
undulator beam waist (geometric)[11,12]	$w_0 \geq \sqrt{2\pi}\sigma_{\mathrm{e}}$
undulator Rayleigh length (geometric)[11–13]	$2z_{\mathrm{R}} \geq L$
undulator pulse duration[11]	$\tau_0 \geq \frac{2L}{c}$
emitted wavelength[14]	$\lambda_{\mathrm{L}} = \frac{\lambda_{\mathrm{W}}}{2(1-\cos\phi)\gamma_0^2}(1 + a_0^2 + \gamma_0^2 \vartheta^2)$
undulator linewidth[11]	$\frac{\Delta\lambda_{\mathrm{W}}}{\lambda_{\mathrm{W}}} \leq 2\Gamma$
undulator intensity fluctuations[11,13]	$\frac{\Delta I_0}{I_0} \leq \frac{2\Gamma}{a_0^2}$
longitudinal intensity variations[11]	$\frac{\Delta z}{z_{\mathrm{R}}} \leq \sqrt{\frac{2\Gamma}{a_0^2}}$
transverse intensity variations[11]	$\frac{\Delta x}{w_0} \leq \sqrt{\frac{\Gamma}{a_0^2}}$
electron peak current[11]	$I_p = 2\pi e c n_{\mathrm{e}} \sigma_{\mathrm{e}}^2$
electron bunch charge	$Q = Ne = I_p \tau_{\mathrm{e}}$
electron density fluctuations	$\frac{\Delta n_{\mathrm{e}}}{n_{\mathrm{e}}} \ll \delta$
1D-limit[15]	$\sigma_{\mathrm{e}} > \sigma_{1\mathrm{D}} \equiv \sqrt{L\lambda_{\mathrm{L}}}$
slippage[15]	$\tau_{\mathrm{e}} > \frac{L}{\lambda_{\mathrm{W}}} \frac{\lambda_{\mathrm{L}}}{c}$
cavity crystal bandwidth[16]	$\tau_{\mathrm{e}} > \frac{1}{\sigma_{\omega,\mathrm{crystal}}}$
normalized emittance (geometric)[11,12]	$\varepsilon_n \leq \frac{\sigma_{\mathrm{e}}^2 \gamma_0}{L}$
normalized emittance (beam divergence)[11,12]	$\varepsilon_n \leq \sigma_{\mathrm{e}} \sqrt{\Gamma}$
transverse coherence[11,17,18]	$0.5 \frac{\gamma_0 \lambda_{\mathrm{L}}}{4\pi} \leq \varepsilon_n \leq 10 \frac{\gamma_0 \lambda_{\mathrm{L}}}{4\pi}$
Fermi limit[19]	$\varepsilon_n > \sigma_{\mathrm{e}} \sqrt{\left(\frac{\lambda_{\mathrm{C}}}{2\pi}\right)^3 \frac{\pi \gamma_0 n_{\mathrm{e}}}{\Gamma}}$
cavity length	$L_{\mathrm{cav}} = \frac{c}{2 f_{\mathrm{rep}}}$
reflectivity[7]	$R = 1 - \frac{\omega_{\mathrm{L}} \tau_{\mathrm{inj}}}{Q}$
cavity length fluctuations	$\frac{\Delta L_{\mathrm{cav}}}{L_{\mathrm{cav}}} \leq 2\Gamma$
outcoupled photons	$n_{\mathrm{out}} = (1-R) n_{\mathrm{st}}$

Table S2. Constraints for a Quantum FEL oscillator experiment in the small-signal regime.

References

1. Kling, P. *et al.* What defines the quantum regime of the free-electron laser? *New J. Phys.* **17**, 123019, DOI: 10.1088/1367-2630/17/12/123019 (2015).
2. Sargent III, M., Scully, M. O. & Lamb Jr., W. E. *Laser Physics* (Addison-Wesley, Reading, 1974).
3. Schleich, W. P. *Quantum Optics in Phase Space* (Wiley-VCH, Weinheim, 2001).
4. Meystre, P. & Sargent III, M. *Elements of Quantum Optics* (Springer Berlin, Heidelberg, 2007).
5. Lugiato, L. A., Scully, M. O. & Walther, H. Connection between microscopic and macroscopic maser theory. *Phys. Rev. A* **36**, 740, DOI: 10.1103/PhysRevA.36.740 (1987).
6. Görtz, R. & Walls, D. Steady state solutions of master equations without detailed balance. *Zeitschrift für Physik B* **25**, 423–427, DOI: 10.1007/BF01315258 (1976).
7. Haken, H. *Light, Volume 2 – Laser Light Dynamics* (North-Holland Physics Publishing, Amsterdam, 1985).
8. Becker, W. & McIver, J. K. Many-particle quantum theory for a class of free-electron devices. *Phys. Reports* **154**, 205–245, DOI: 10.1016/0370-1573(87)90068-8 (1987).
9. Bambini, A. & Renieri, A. The free electron laser: A single-particle classical model. *Lett. Nuovo Cimento* **21**, 399–404, DOI: 10.1007/BF02762613 (1978).
10. Bambini, A., Renieri, A. & Stenholm, S. Classical theory of the free-electron laser in a moving frame. *Phys. Rev. A* **19**, 2013 – 2025, DOI: 10.1103/PhysRevA.19.2013 (1979).
11. Debus, A., Steiniger, K., Kling, P., Carmesin, C. M. & Sauerbrey, R. Realizing quantum free-electron lasers: a critical analysis of experimental challenges and theoretical limits. *Phys. Scr.* **94**, 074001, DOI: 10.1088/1402-4896/aaf951 (2019).
12. Steiniger, K. *et al.* Optical free-electron lasers with traveling-wave Thomson-scattering. *J. Phys.* **47**, 234011, DOI: 10.1088/0953-4075/47/23/234011 (2014).
13. Bonifacio, R., Piovella, N., Cola, M. M. & Volpe, L. Experimental requirements for X-ray compact free electron lasers with a laser wiggler. *Nucl. Instrum. & Methods A* **577**, 745–750, DOI: https://doi.org/10.1016/j.nima.2007.03.024 (2007).
14. Ride, S. K., Esarey, E. & Baine, M. Thomson scattering of intense lasers from electron beams at arbitrary interaction angles. *Phys. Rev. E* **52**, 5425–5442, DOI: 10.1103/PhysRevE.52.5425 (1995).
15. Schmüser, P., Dohlus, M. & Rossbach, J. *Ultraviolet and Soft X-Ray Free-Electron Lasers* (Springer, Heidelberg, 2008).
16. Lindberg, R. R., Kim, K.-J., Shvyd'ko, Y. & Fawley, W. M. Performance of the x-ray free-electron laser oscillator with crystal cavity. *Phys. Rev. ST Accel. Beams* **14**, 010701, DOI: 10.1103/PhysRevSTAB.14.010701 (2011).
17. Saldin, E. L., Schneidmiller, E. A. & Yurkov, M. V. Coherence properties of the radiation from X-ray free electron laser. *Opt. Commun.* **281**, 1179–1188, DOI: https://doi.org/10.1016/j.optcom.2007.10.044 (2008).
18. Saldin, E. L., Schneidmiller, E. A. & Yurkov, M. V. Output power and degree of transverse coherence of X-ray free electron lasers. *Opt. Commun.* **281**, 4727–4734, DOI: https://doi.org/10.1016/j.optcom.2008.05.033 (2008).
19. Bonifacio, R., Piovella, N. & Robb, G. R. M. The quantum free electron laser: A new source of coherent, short-wavelength radiation. *Fortschr. Phys.* **57**, 1041–1051, DOI: 10.1002/prop.200900097 (2009).
